# Nanovesicles from Organic Agriculture-Derived Fruits and Vegetables: Characterization and Functional Antioxidant Content

**DOI:** 10.3390/ijms22158170

**Published:** 2021-07-29

**Authors:** Mariantonia Logozzi, Rossella Di Raimo, Davide Mizzoni, Stefano Fais

**Affiliations:** Department of Oncology and Molecular Medicine, Istituto Superiore di Sanità, Viale Regina Elena 299, 00161 Rome, Italy; rossella.diraimo@iss.it (R.D.R.); davide.mizzoni@iss.it (D.M.)

**Keywords:** plant-derived nanovesicles, organic agriculture, antioxidants, natural bioactives

## Abstract

Dietary consumption of fruits and vegetables is related to a risk reduction in a series of leading human diseases, probably due to the plants’ antioxidant content. Plant-derived nanovesicles (PDNVs) have been recently receiving great attention regarding their natural ability to deliver several active biomolecules and antioxidants. To investigate the presence of active antioxidants in fruits, we preliminarily analyzed the differences between nanovesicles from either organic or conventional agriculture-derived fruits, at equal volumes, showing a higher yield of nanovesicles with a smaller size from organic agriculture-derived fruits as compared to conventional ones. PDNVs from organic agriculture also showed a higher antioxidant level compared to nanovesicles from conventional agriculture. Using the PDNVs from fruit mixes, we found comparable levels of Total Antioxidant Capacity, Ascorbic Acid, Catalase, Glutathione and Superoxide Dismutase 1. Finally, we exposed the nanovesicle mixes to either chemical or physical lytic treatments, with no evidence of effects on the number, size and antioxidant capacity of the treated nanovesicles, thus showing a marked resistance of PDNVs to external stimuli and a high capability to preserve their content. Our study provides for the first time a series of data supporting the use of plant-derived nanovesicles in human beings’ daily supplementation, for both prevention and treatment of human diseases.

## 1. Introduction

The beneficial effects of food and its bioactive components on human health and disease are widely recognized [1,2,3,4,5]; in fact, this knowledge covers a huge part of the pharmaceutical market [6,7]. Unfortunately, the vast majority of the food-derived products come from chemical extractions, which highly impairs their bioavailability [5]. Recently, it has been reported that food-derived nanovesicles can enhance the efficacy of natural compounds through a marked improvement in their bioavailability, probably due to the natural protection exerted by the nanovesicles on their bioactive content [8]. Plant-derived nanovesicles (PDNVs) are small vesicles surrounded by a lipid-enriched membrane and containing different natural compounds, including vitamins, antioxidants, proteins, nucleic acids and other metabolites. All these bio-compounds have shown to maintain their biological activities when uploaded to target cells. The mean size of the nanovesicles ranges between 250 nm (grapefruit and ginger EVs) [8,9,10] and 400 nm (grape) [9,11]. This may depend on the fruit or vegetables under observation; for instance, nanovesicles isolated from carrots are more heterogeneous, with sizes ranging from 100 nm to 1000 nm [9,12]. Only recently we have collected more information on the biogenesis of plant-derived nanovesicles [13]. This first report showed, by TEM analysis, the presence of Multivesicular Bodies (MVBs) on barley leaf cells attacked by a fungus. More recently, it has been postulated that MVBs participate in the intake of nutrients from apoplast, and of damaged membranes and deleterious materials originating from the oxidative microburst [14]. PDNVs play several crucial roles in plant cell homeostasis, including unconventional plant cell wall remodeling [15] and plant defense [16,17,18]. In fact, these nanovesicles have evolved in plants as a way to communicate between both plant cells and other plants or animals [12,16]. Nanovesicles were found both in the xylem and phloem of woody plants, supporting their role in the storage and transport of endoglucanases [19], essential for plant growth [20]. Further studies have supported the role of PDNVs in the modulation of first-line innate responses when plants are attacked by pathogens [11,16,17,18], by also transferring non-coding RNAs to pathogens [12,16] and in regulating gene expression over long distances [21]. Production of vesicles is increased to contrast biotic stresses during pathogen infection [18,22,23]. Plant-derived vesicles were found in xylem wash fluid, but how they cross the plant wall remains unclear [24,25].

The constitutive properties of PDNVs make them suitable for clinical application, including delivery through internalization, low immunogenicity, stability in the gastrointestinal tract and the ability to cross the Blood–Brain Barrier [10,26,27]. Some recent studies have been focused on the interaction between nanovesicles and intestinal tissue. For example, nanovesicles from ginger, grapefruit, grape, lemon and broccoli may have a role in prevention of inflammatory bowel disease, by both blocking the damaging factors and improving the expression of the healing factors [10,11,12,28,29,30,31]. Moreover, ginger-derived nanovesicles both induce the expression of detoxifying genes and inhibit ROS accumulation in the liver injured by alcohol [32]. Strawberry- and lemon-derived vesicles exert a potent antioxidant effect preventing oxidative stress in Human Mesenchymal Cells [33,34]. Intraperitoneal administration of honey-derived nanoparticles alleviates inflammation and liver damage in mice with liver injury [35]. Moreover, it has been shown that PDNVs exert anti-cancer effects in melanoma and lung carcinoma-derived cell lines [29,36,37]. Some clinical trials are currently in progress with the purpose to evaluate the ability of PDNVs to either prevent or treat human diseases, e.g., a study is ongoing with the aim to evaluate the effectiveness of PDNVs in reducing insulin resistance (NCT03493984) [38], while another study is investigating the ability of PDNVs in preventing the side effects of chemotherapy (NCT01668849) [39]. They can also be used as drug delivery system in combination with siRNAs and miRNAs [26,40].

A wide panel of artificial nanovesicles are still under investigation, while their use is limited by their low production scale and the need to test each component in vivo before clinical application. PDNVs are excellent candidates as nanodelivery carriers since they are totally natural vesicles, intrinsically made of sustainable and biodegradable materials, and with the potential to be isolated in large amounts [41,42]. Moreover, PDNVs have shown a higher uptake efficiency as compared to artificial nanoparticles, such as liposome formulations [40]. Another plus point for the clinical application of plant nanovesicles is they can be obtained by organic agriculture, which is without pesticides and chemical fertilizers. In fact, our group has demonstrated that human exosomes actively participate in the scavenging activity of unwanted or toxic materials, such as gold nanoparticles [43]. By analogy, plant nanovesicles may be involved in the elimination of toxic materials present in the environment by concentrating the toxics into the PDNVs. Based on the above background, in this study, we first wanted to evaluate the differences between the nanovesicles isolated from organic and conventional fruits in terms of both yield and Total Antioxidant Capacity. A further set of experiments was aimed at measuring the level of the effective bioactive form of the analyzed molecules in plant-derived nanovesicles, by using specific ELISA colorimetric assays. Thereby we characterized the antioxidant content of the nanovesicles isolated from two fruit mixes in terms of Total Antioxidant Capacity and single antioxidants such as Catalase and Superoxide Dismutase 1, Glutathione and Ascorbic Acid. We finally evaluated the resistance of the membranes to both chemical and physical lysis, analyzing the number, size and Total Antioxidant Capacity of the nanovesicles.

## 2. Results

### 2.1. Characterization of Nanovesicles from Organic and Conventional Agriculture

**Figure 1:**
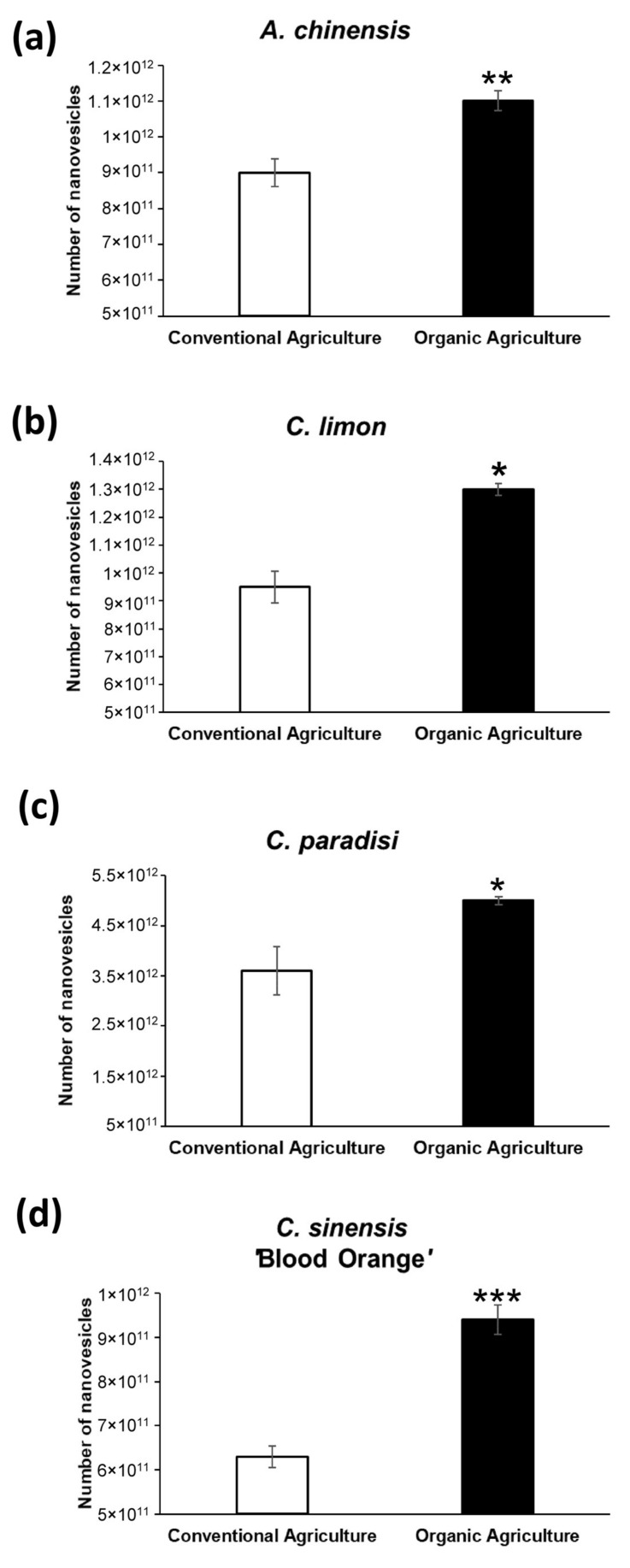
Quantification of conventional and organic agriculture-derived nanovesicles through Nanoparticle Tracking Analysis (NTA): (**a**) quantification of nanovesicles isolated from kiwi (*A. chinensis*); (**b**) quantification of nanovesicles isolated from lemon (*C. limon*); (**c**) quantification of nanovesicles isolated from grapefruit (*C. paradisi*); (**d**) quantification of nanovesicles isolated from blood orange (*C. sinensis* ‘Blood Orange’). Data are expressed as the mean ± SE. *: *p* < 0.05; **: *p* < 0.001; ***: *p* < 0.0001.

Organic production is not simply the avoidance of conventional chemical inputs but relies on ecological processes and cycles adapted to local conditions, aiming to maintain biological diversity and produce food in an eco-sustainable way. It is thus conceivable that the farming system (organic or conventional) can influence the characteristics of the crop and consequently the amount and content of isolated vesicles. Thereby, starting from the same amount of fruit extracts, we isolated nanovesicles from four fruits: kiwi (*A. chinensis*), lemon (*C. limon*), grapefruit (*C. paradisi*) and blood orange (*C. sinensis* ‘Blood Orange’), both from organic and conventional agriculture. To investigate the differences between the isolated nanovesicles, we analyzed them through Nanoparticle Tracking Analysis (NTA) to determine their number and size, and through a colorimetric ELISA kit to detect and quantify their Total Antioxidant Capacity. As shown in Figure 1 and Table 1, nanovesicles derived from organic agriculture were many more compared to the conventional ones. In particular, from organic fruits we isolated 22% (*A. chinensis*, *p* < 0.001), 36% (*C. limon p* < 0.05), 39% (*C. paradisi*, *p* < 0.05) and 49% (*C. sinensis* ‘Blood Orange’, *p* < 0.0001) more vesicles compared to conventional fruits. Significant differences were also observed in the size of the nanovesicles: nanovesicles isolated from organic agriculture-derived fruits were smaller compared to vesicles from conventional fruits (*p* < 0.05) (Table 1).

**Figure 2:**
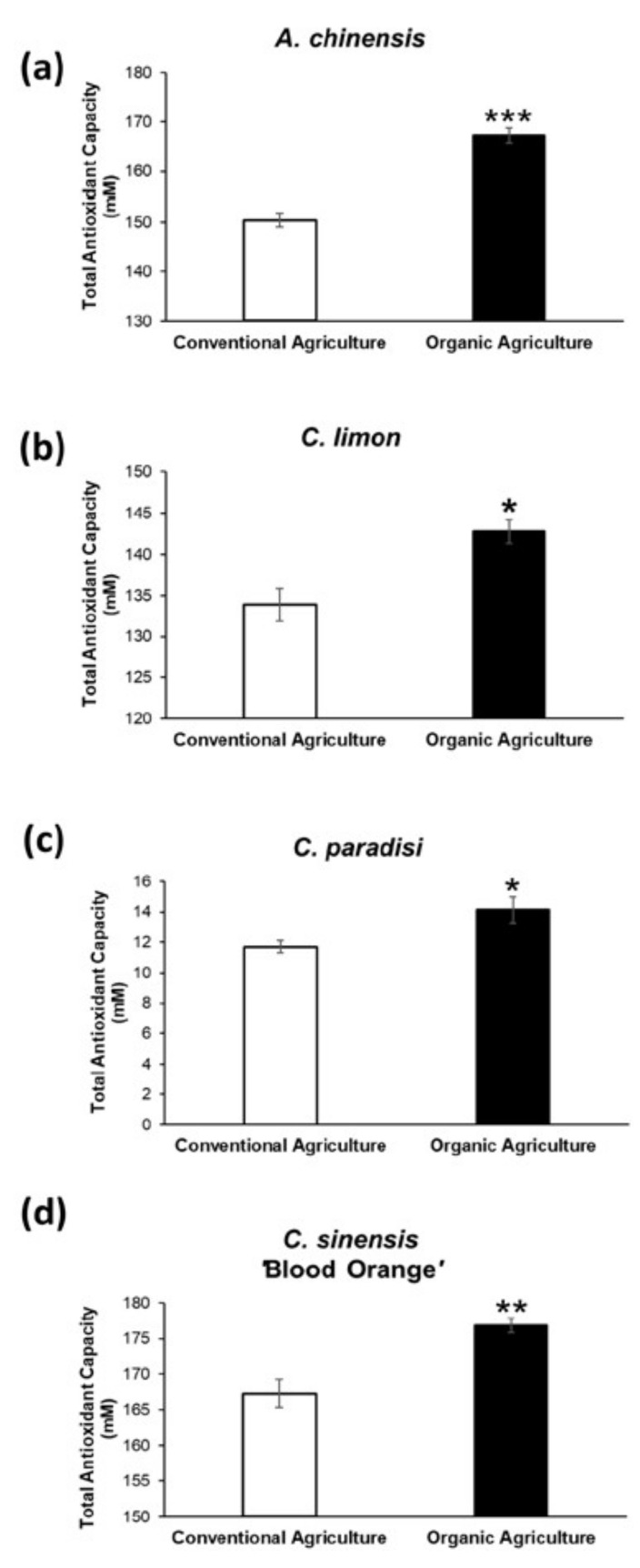
Determination of the Total Antioxidant Capacity (TAC) of organic and conventional agriculture-derived nanovesicles: (**a**) Total Antioxidant Capacity of the nanovesicles isolated from kiwi (*A. chinensis*); (**b**) Total Antioxidant Capacity of the nanovesicles isolated from lemon (*C. limon*); (**c**) Total Antioxidant Capacity of the nanovesicles isolated from grapefruit (*C. paradisi*); (**d**) Total Antioxidant Capacity of the nanovesicles isolated from blood orange (*C. sinensis* ‘Blood Orange’). Data are expressed as the mean ± SE. *: *p* < 0.05; **: *p* < 0.001; ***: *p* < 0.0001.

To further investigate the differences between the vesicles isolated from organic agriculture fruits and conventional ones, we evaluated the antioxidant content. In Figure 2 are reported the Total Antioxidant Capacities of the various analyzed samples. Total Antioxidant Capacity (TAC) is not only the sum of the antioxidant capacities of individual bioactive compounds but also derived from the synergistic effects between the bioactive compounds, metals and other food constituents [44]. The test used can detect both hydrophilic (i.e., vitamin C and glutathione) and hydrophobic antioxidants (i.e., Vitamin E). Like the nanovesicle number quantification, the antioxidant content of the PDNVs isolated from the organic fruits were higher compared to the PDNVs from conventional fruits. In detail, the Total Antioxidant Capacities in the organic agriculture-derived nanovesicles were 11% (*A. chinensis*, *p* < 0.0001), 7% (*C. limon, p* < 0.05), 21% (*C. paradisi*, *p* < 0.05) and 6% (*C. sinensis* ‘Blood Orange’, *p* < 0.001) higher than TAC in conventional nanovesicles.

### 2.2. Characterization and Antioxidant Content of Nanovesicles Isolated from Two Different Fruit Mixes

**Figure 3:**
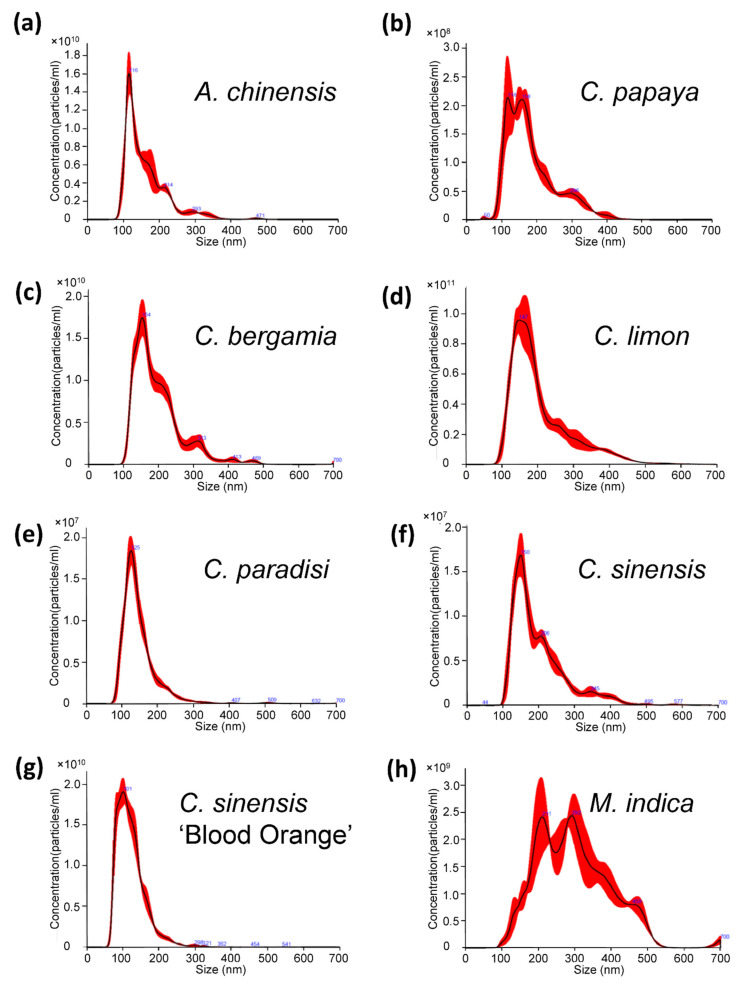
Distribution of organic agriculture-derived nanovesicles analyzed through NTA: (**a**) distribution of the nanovesicles isolated from kiwi (*A. chinensis*); (**b**) distribution of the nanovesicles isolated from papaya (*C. papaya*); (**c**) distribution of the nanovesicles isolated from bergamot (*C. bergamia*); (**d**) distribution of the nanovesicles isolated from lemon (*C. limon*); (**e**) distribution of the nanovesicles isolated from grapefruit (*C. paradisi*); (**f**) distribution of the nanovesicles isolated from orange (*C. sinensis*); (**g**) distribution of the nanovesicles isolated from blood orange (*C. sinensis* ‘Blood Orange’); (**h**) distribution of the nanovesicles isolated from mango (*M. indica*).

After determining the differences between the nanovesicles from organic and conventional fruit, we decided to study the content of PDNVs isolated from organic fruit mixes. Starting from the evidence that the antioxidant activity is increased when a combination of fruits and/or plants is used [45,46,47], it is plausible that the antioxidant activity is also increased in the isolated nanovesicles. For this purpose, we prepared two different fruit mixes: Mix 1 was composed of kiwi (*A. chinensis*), orange (*C. sinensis*), blood orange (*C. sinensis* ‘Blood Orange’), lemon (*C. limon*) and papaya (*C. papaya* L.); and Mix 2 of bergamot (*C. bergamia*), grapefruit (*C. paradisi*), orange (*C. sinensis*), blood orange (*C. sinensis* ‘Blood Orange’), lemon (*C. limon*) and mango (*M. indica*). First of all, we isolated nanovesicles from individual fruit extracts and analyzed them through NTA (Figure 3). All the samples analyzed have the typical sizes of extracellular vesicles: *A. chinensis* 161 ± 0.6 nm (Figure 3a), *C. papaya* 172.8 ± 12.8 nm (Figure 3b), *C. bergamia* 190.2 ± 7.2 nm (Figure 3c), *C. limon* 194 ± 3.1 nm (Figure 3d), *C. paradisi* 141.7 ± 5.9 nm (Figure 3e), *C. sinensis* 154 ± 6.3 nm (Figure 3f), *C. sinensis* ‘Blood orange’ 125.3 ± 2.7 (Figure 3g) and *M. indica* 285 ± 11.5 nm (Figure 3h).

**Figure 4:**
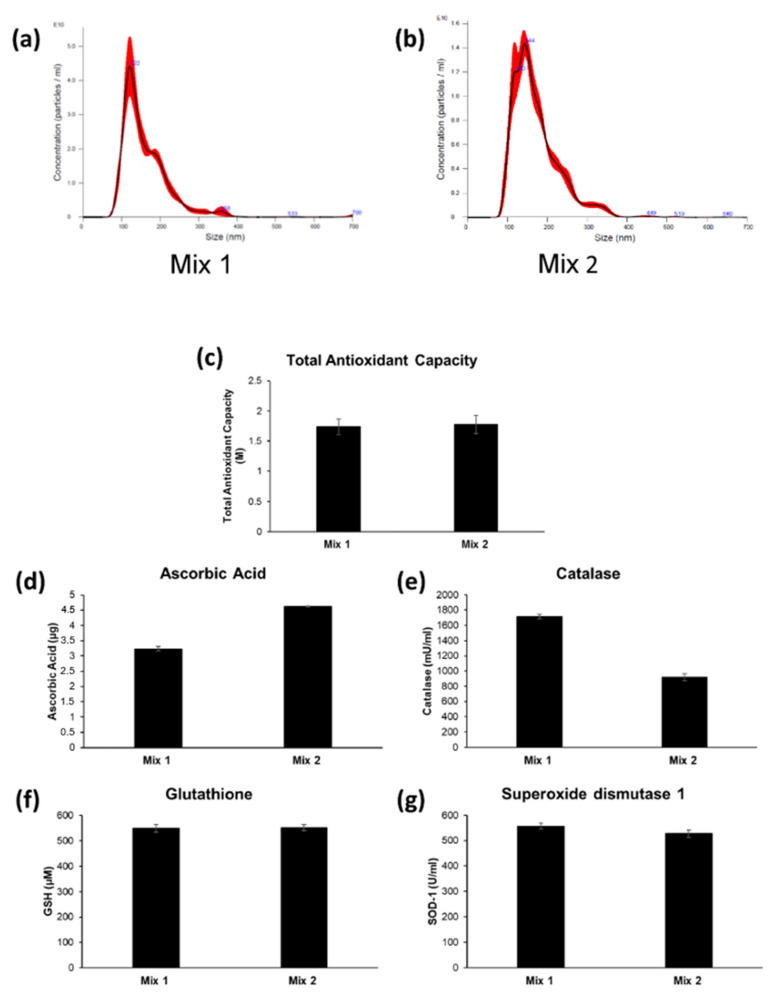
Characterization of antioxidants in organic-derived nanovesicles: (**a**) distribution of the nanovesicles isolated from Mix 1 analyzed through NTA; (**b**) distribution of the nanovesicles isolated from Mix 2 analyzed through NTA; (**c**) quantification of the Total Antioxidant Capacity in nanovesicles isolated from Mix 1 and Mix 2; (**d**) quantification of the Ascorbic Acid in nanovesicles isolated from Mix 1 and Mix 2; (**e**) quantification of the Catalase activity in nanovesicles isolated from Mix 1 and Mix 2; (**f**) quantification of the Glutathione in nanovesicles isolated from Mix 1 and Mix 2; (**g**) quantification of the Superoxide Dismutase 1 activity in nanovesicles isolated from Mix 1 and Mix 2. Data are expressed as the mean ± SE.

Then we analyzed the distribution of the nanovesicles isolated from fruit mixes through Nanoparticle Tracking Analysis and the results are reported in Figure 4a,b. Although deriving from different fruits, the isolated nanovesicles have a fairly homogeneous distribution, with no signs of damage even after the ultracentrifugation rounds. In fact, the nanovesicles’ sizes for Mix 1 and Mix 2 were, respectively, 165.9 ± 8.9 nm and 176.3 ± 10.8 nm, confirming the presence of the vesicles within the typical range of nanovesicles. After the quality assessment, in terms of both number and size of the nanovesicles through NTA, we quantified the antioxidant content of a defined number of nanovesicles though ELISA assay. We first measured the Total Antioxidant Capacity (TAC), which includes the cumulative and synergistic action of all antioxidants present in the analyzed samples, thus providing a measure of the entire antioxidant content in our nanovesicle preparation, rather than a mere sum of each single, measurable antioxidant. The results showed that the nanovesicle preparation derived from both fruit mixes contained very high levels of TAC: respectively, 1.74 ± 0.13 M for Mix 1 and 1.78 ± 0.15 M for Mix 2. Thus, we stepped ahead by measuring the single antioxidants within our nanovesicle preparations. Within the same preparations, both enzymatic antioxidants, such as Catalase and Superoxide dismutase 1 (SOD-1), and non-enzymatic antioxidants, such as Glutathione (GSH) and Ascorbic Acid, were measurable (Figure 4). Ascorbic Acid in plants plays predominantly a protective role by scavenging reactive oxygen species, but it is also an important cofactor for several enzymes involved in regulating photosynthesis, hormone biosynthesis and regenerating other antioxidants [48,49,50]. We were able to measure virtually equal amounts of Ascorbic Acid in both mixes: 3.23 ± 0.07 µg for Mix 1-derived nanovesicles and 4.61 ± 0.02 µg for Mix 2-derived nanovesicles, respectively (Figure 4d). Catalase in plants is a crucial enzyme for survival under metal stress, besides having a fundamental role in the scavenging of reactive oxygen species [51,52]. In nanovesicles isolated from both mixes, we determined high levels of Catalase activity: respectively, 1713 ± 30 mU/mL and 920 ± 45 mU/mL for Mix 1 and Mix 2 (1 unit of Catalase activity corresponds to amount of Catalase that decomposes 1.0 µmol of H_2_O_2_ per minute at pH 4.5 at 25 °C) (Figure 4e). Glutathione is considered a strong non-enzymatic antioxidant, which participates both directly and indirectly in the detoxification of ROS, and as cofactor of several biochemical reactions, including signal transduction [53,54]. We found equal Glutathione concentrations in the two mixes (549 ± 15 µM for Mix 1 and 551.9 ± 12 µM for Mix 2). Finally, we investigated Superoxide Dismutase 1 activity in our nanovesicles’ preparations. Superoxide Dismutase is known as the first line of defense against oxidative stresses in plants and plays pivotal role in scavenging the reactive oxygen species generated from both metabolic processes and under abiotic stresses [55,56,57]. Again, the two nanovesicle mixes showed highly comparable amounts of SOD-1 (557 ± 13 U/mL for Mix 1 and 527 ± 14 U/mL for Mix 2). This set of results showed that nanovesicles purified from either different single fruits or mixes of fruit extracts both exert a very potent Total Antioxidant Capacity (TAC) and contain single antioxidants that are clearly measurable as single bioactives. 

### 2.3. Nanovesicles Are Resistant to Chemical Lysis

**Figure 5:**
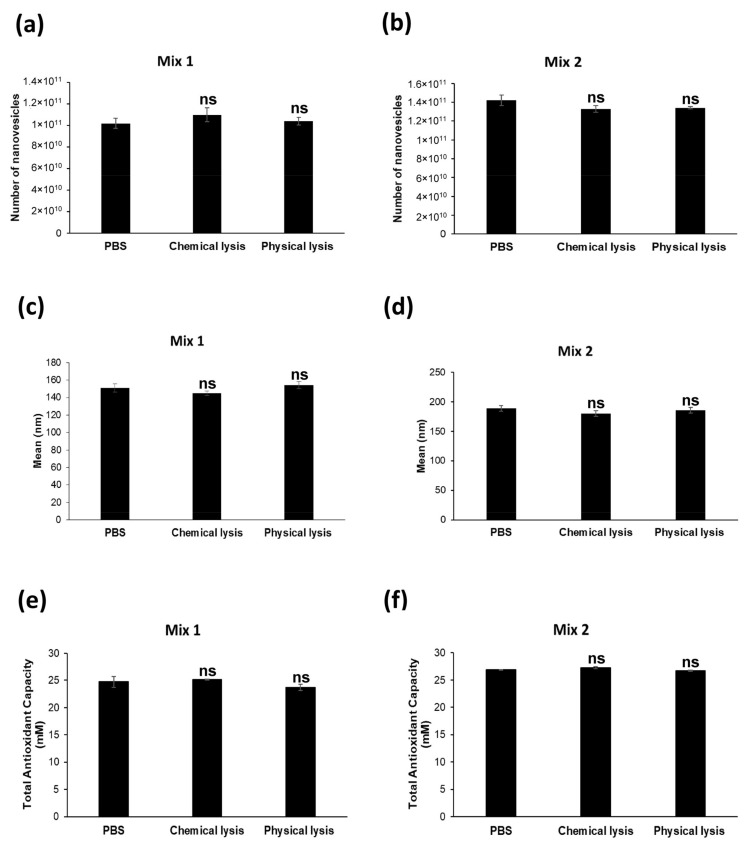
Evaluation of nanovesicle resistance to lysis: (**a**) comparison of the number of nanovesicles isolated from Mix 1 in PBS (not lysed), after chemical lysis (CHAPS buffer) and physical lysis (ultrasonic bath sonicator), analyzed through NTA; (**b**) comparison of the number of nanovesicles isolated from Mix 2 in PBS (not lysed), after chemical lysis (CHAPS buffer) and physical lysis (ultrasonic bath sonicator), analyzed through NTA; (**c**) comparison of the mean size of the nanovesicles isolated from Mix 1 in PBS (not lysed), after chemical lysis buffer (CHAPS buffer) and physical lysis (ultrasonic bath sonicator), analyzed through NTA; (**d**) comparison of the mean size of the nanovesicles isolated from Mix 2 in PBS (not lysed), after chemical lysis buffer (CHAPS buffer) and physical lysis (ultrasonic bath sonicator), analyzed through NTA; (**e**) comparison of the Total Antioxidant Capacity in nanovesicles isolated from Mix 1 in PBS (not lysed), after chemical lysis (CHAPS buffer) and physical lysis (ultrasonic bath sonicator), analyzed through a colorimetric ELISA kit; (**f**) comparison of the Total Antioxidant Capacity in nanovesicles isolated from Mix 2 in PBS (not lysed), after chemical lysis (CHAPS buffer) and physical lysis (ultrasonic bath sonicator), analyzed through a colorimetric ELISA kit. Data are expressed as the mean ± SE. ns: *p* > 0.05.

It is well known that plant-derived nanovesicles are highly stable at different conditions and resistant to digestion by gastric pepsin solution, pancreatic enzymes and bile [40]. For the purpose of evaluating the resistance of the nanovesicles to lytic substances, we exposed our nanovesicle preparations to either chemical lysis, through a lysis buffer commonly used for protein extraction or physical lysis, or through an ultrasonic bath sonicator. Following the two alternative treatments, we compared the number, mean size and Total Antioxidant Capacity in either the lysed or non-lysed nanovesicles. As reported in Figure 5, the results obtained by NTA did not show statistically significant differences between all the analyzed conditions. In detail, for Mix 1, the number of lysed nanovesicles was 1.1 × 10^11^ ± 6.45 × 10^9^ (chemical lysis) and 1.04 × 10^11^ ± 3.64 × 10^9^ (physical lysis), as compared to the non-lysed initial vesicles (1.02 × 10^11^ ± 4.72 × 10^9^) (ns: *p* > 0.05) (Figure 5a). For Mix 2, the number of nanovesicles that underwent the lytic treatments were 1.33 × 10^11^ ± 3.66 × 10^9^ after chemical lysis, and 1.34 × 10^11^ ± 1.29 × 10^9^ after physical lysis as compared to non-lysed nanovesicles (1.42 × 10^11^ ± 5.62 × 10^9^) (ns: *p* > 0.05).

Through NTA analysis, we also determined the mean size of the lysed and non-lysed nanovesicles. Comparably to the number, we found no significant differences between the lysed and non-lysed samples for the isolated nanovesicles from both fruit mixes (Mix 1: non-lysed nanovesicles 151.2 ± 4.6 nm; after chemical lysis 145.1 ± 2.4 nm; and after physical lysis 154.5 ± 4.7 nm; ns: *p* > 0.05. Mix 2: non-lysed nanovesicles 188.6 ± 4.5 nm; after chemical lysis 179.9 ± 5.0 nm; and after physical lysis 185.4 ± 5.1 nm; ns: *p* > 0.05) (Figure 5c,d). Taking together, these data showed clearly that our fruit-derived nanovesicles were fully resistant to both chemical and physical lysis, the number and size of the treated nanovesicles remaining virtually identical to the untreated samples, in turn suggesting the high level of resistance to degradative stimuli of the plant-derived nanovesicles. To complete this set of experiments, in the same samples we compared the Total Antioxidant Capacity between nanovesicles undergoing, or not, lytic treatments. Similarly to the results obtained by analyzing the number and size, the nanovesicles showed a highly comparable TAC concentration, independent of the treatment used: for Mix 1 24.7 ± 1.0 mM without treatment, 25.1 ± 0.1 mM following CHAPS buffer treatment and 23.7 ± 0.6 mM after lysis in an ultrasonic bath sonicator (ns: *p* > 0.05) (Figure 5e); we obtained comparable results with Mix 2 as well (26.9 ± 0.04 mM in PBS, 27.3 ± 0.2 mM after chemical lysis and 26.7 ± 0.1 mM after physical lysis) (ns: *p* > 0.05) (Figure 5f).

This set of results showed that lytic treatments did not affect the integrity of the plant-derived nanovesicles, not only in terms of number and size but in their antioxidant content as well.

## 3. Discussion

The correlation between dietary consumption of antioxidants from fruits and vegetables and the increase in antioxidant levels in treated subjects have been widely studied [58,59,60]. Moreover, we also know that a diet mostly based on fruits and vegetables, or their derivates, is associated with a reduced risk of diabetes, cardiovascular diseases, age-related diseases, and cancer [60,61,62,63,64,65,66,67,68,69]. On the other hand, the majority of suppliers derive antioxidants by either chemical extraction or synthetic phytochemical procedures, which do not show the same effectiveness as plant derivates. From our data and the data of other authors, it appears conceivable that the different bioactives contained in plants may be complexed and, in a way, protected from oxidation and degradation, thus rendering them more powerful than single bioactives. Another crucial issue is the level of bioavailability that is indeed low or very low as far as the standard suppliers are concerned [5,42]. Nanovesicles can be isolated from different fruits and vegetables [41,70,71] and they could well overcome the limitations of current bioproducts. Lastly, plants represent green, sustainable and renewable sources of nanovesicles [72,73], and this can ensure a constant and never-ending production, in turn providing bioproducts that are immediately available at low dosages, stable and more effective than the current products, and therefore more suitable for clinical use and the market. 

In this study, we first obtained nanovesicles isolated from organic agriculture-derived fruits, in turn evaluating the potential differences between the nanovesicles isolated from fruits deriving from either organic or conventional agriculture. The fundamental difference between these two farming systems is the total absence of synthetic pesticides in organic agriculture, in order to limit consumers’ exposure to pesticide residues in fruits and vegetables [74,75]. A quantitative analysis by NTA has shown that at an equal volume of fruit juice we obtained more nanovesicles from the juice of an organic agriculture source as compared to the juice from intensive agriculture. Moreover, nanovesicles from the juice of organic agriculture showed higher Total Antioxidant Capacity as compared to conventional agriculture-derived nanovesicles. These data appear highly reasonable and conceivable inasmuch as these different agriculture practices may have a strong impact on a plant’s metabolism, leading to differences in crop composition, also reflecting on the fruit and vegetable harvesting and therefore on the yield of the internal components of the plants, including nanovesicles [75,76]. Moreover, natural fertilizers in organic farming affect the profile of the secondary metabolites in plant tissues by changing the protein expression [76,77,78,79]. 

In this study, we also measured the antioxidant content in nanovesicles isolated from either single fruits or a mix fruit extracts. After a careful characterization of both the distribution and size of the nanovesicles derived from the two sets of fruit, we first measured the Total Antioxidant Capacity (TAC) (i.e., the amount of free radicals scavenged by a test solution), which is typically used for antioxidant determination in biological samples [80,81,82,83]. Actually, TAC depends on the synergy, in terms of redox interaction, between the different bioactives present in the various foods [84,85]. We thus decided to evaluate the levels of single antioxidants in nanovesicles isolated form different fruits. We knew that some constitutive bioactives of plants, such as Catalase, Glutathione (GSH), Superoxide Dismutase 1 (SOD 1) and Ascorbic Acid, are all fully able to scavenge H_2_O_2_ and reactive oxygen species (ROS), generated by a series of cellular processes, including mitochondrial electron transport, β-oxidation of the fatty acids and photorespiratory oxidation [49,53,54,55,56,86,87]. Moreover, the abovementioned antioxidants play a crucial role in plant defense, aging and senescence [86], plant tolerance to abiotic stresses and toxic metal stress [53]. For instance, GSH scavenges free radicals and other reactive oxygen species directly or indirectly through enzymatic reactions [54,87]. Based on this background, we were able to detect and quantify several antioxidants in nanovesicles isolated from organic fruit mixes, including Catalase, GSH, SOD 1 and Ascorbic Acid. The evidence that the various antioxidants are contained and fairly measurable in the fruit’s nanovesicles suggests that when ingested within nanovesicles, the antioxidants may remain stably bioavailable, being protected from quick oxidation but also from lysis and enzymatic digestion in gastric and intestinal solutions [9,40]. This suggests the potential use of vegetables-derived derived nanovesicles as a therapeutic agent in oxidative stress- and age-related diseases [52,57]. The results of this study confirm that plant-derived nanovesicles represent an excellent nanodelivery system for constitutively expressed natural compounds, thanks to their sizes, safety, biocompatibility, and stability [42,88]. For example, grapefruit nanovesicles are highly stable in solutions mimicking gastric and intestinal conditions [40]. In fact, our results have also shown that both chemical and physical lysis did not affect the nanovesicles’ distribution and antioxidant content, further supporting the high potential of fruit and vegetable-derived nanovesicles for therapeutic use. 

All in all, the results of our study provide for the first time a series of critical evidence on the potential use of plant-derived nanovesicles in human beings’ daily supplementation. First, they can be obtained and analyzed in both a quantitative and qualitative way. There are substantial, both quantitative and qualitative, differences between the nanovesicles obtained from fruits deriving from organic agriculture as compared to those obtained from conventional agriculture. The bioactives contained in plant-derived nanovesicles are stable and complexed between them within the nanovesicles, thus allowing a more powerful and combined antioxidant effect. The bioactives are fairly stable within the nanovesicles, thus allowing a long-standing bioavailability. 

## 4. Materials and Methods

### 4.1. Fruit Material

Kiwi (*A. chinensis*), orange (*C. sinensis*), blood orange (*C. sinensis* ‘Blood Orange’), lemon (*C. limon*), papaya (*C. papaya* L.) and mango (*M. indica*) were purchased from several farms with organic farming certification and from conventional farms, under the same conditions (season, climate, area of cultivation and degree of ripeness). The fruits were washed with water and bicarbonate, peeled, and extracted with a fruit juice extractor. Fruit juices were stored at −80 °C. 

### 4.2. Nanovesicles Isolation

Fruit juices were centrifuged at 500× *g* × 10 min; the supernatants were filtered with 100 µm filters and serially centrifugated at 2000× *g* for 20 min to eliminate cell debris and then at 15,000× *g* for 30 min to eliminate the fraction enriched in microvesicles. The supernatants were subsequently ultracentrifuged in a Sorvall WX Ultracentrifuge Series (Thermo Fisher Scientific) at 110,000× *g* for 1 h 30 min to collect the nanovesicles. The pellet was resuspended in an appropriate buffer for downstream analyses. 

### 4.3. Nanoparticle Tracking Analysis

Nanoparticle Tracking Analysis (NTA) from Malvern (NanoSight NS300, Worcestershire, UK) was used for the measurement of size distribution and concentration of extracellular vesicle samples in the liquid suspension. Five videos of typically 60 s duration were taken. Data were analyzed using the NTA 3.0 software (Malvern Instruments), which was optimized to first identify and then track each particle on a frame-by-frame basis. The Brownian motion of each particle was tracked using the Stokes–Einstein equation: D° = kT/6πηr, where D° is the diffusion coefficient, kT/6πηr = f0 is the frictional coefficient of the particle, for the special case of a spherical particle of radius r moving at a uniform velocity in a continuous fluid of viscosity η, k is Boltzmann’s constant, and T is the absolute temperature.

### 4.4. Total Antioxidant Power Assay (PAO Test Kit)

Detection and quantification of Total Antioxidant Capacity were performed in nanovesicles using a colorimetric assay: PAO Test kit for Total Antioxidant Capacity (JaICA, Shizuoka, Japan). The assay can detect not only hydrophilic antioxidants, such as Vitamin C and glutathione, but can also detect hydrophobic antioxidants, such as Vitamin E. The determination of the antioxidant power was carried out using a reduction of cupric ion (Cu^++^ to Cu^+^). Briefly, samples were incubated for 3 min at room temperature with a Cu^++^ solution, and the Cu^++^ was reduced by antioxidants to form Cu^+^ that reacts with a chromatic solution (bathocuproine), and can be detected by absorbance at a wavelength of 480 to 490 nm. Antioxidant capacity can be calculated from the Cu^+^ formed. Absorbance was recorded at 490 nm.

### 4.5. Ascorbic Acid Assay

Detection and quantification of Ascorbic Acid in nanovesicles were performed using a fluorometric Ascorbic Acid Assay Kit (Sigma-Aldrich, St. Louis, MO, USA). Samples were diluted in ascorbic acid buffer in a 96-well plate and subsequently to each well was added a catalyst and then reaction mix (the reaction mix is composed of an ascorbic acid buffer, ascorbic acid probe and ascorbic acid enzyme mix). After 5 min of incubation, fluorescence was read in a microplate reader at Ex/Em = 535/587 nm.

### 4.6. Catalase Activity Assay

For the Catalase Activity Assay (Abcam, Cambridge, UK), a fluorometric kit was used for detection and quantification of the Catalase activity in fruit-derived nanovesicles. Briefly, samples resuspended in PBS were loaded in a 96-well plate; a stop solution was added in the control samples and incubated for 5 min at 25 °C to inhibit the Catalase activity. Catalase reaction mix (with H_2_O_2_) was added to both the control and high control samples for 30 min at 25 °C. The reaction in the high control samples and standard samples was stopped with the stop solution, the developer was added to all wells and after 10 min the fluorescence was read at Ex/Em = 535/587 nm on a microplate reader (Promega, Madison, WI, USA). Data were analyzed using the manufacturer’s instructions. One unit of Catalase corresponds to the amount of Catalase that will decompose 1 µmol of H_2_O_2_ per minute at pH 4.5 at 25 °C.

### 4.7. Reduced Glutathione (GSH) Detection and Quantification Assay

The Glutathione Colorimetric Detection Kit (Thermo Fisher, Waltham, MA, USA), a colorimetric assay, was used for detection and quantification of reduced glutathione (GSH) levels in plasma preparations. Detection reagent and reaction mixture (NADPH and glutathione reductase) were added to samples and after 20 min of incubation at room temperature, the optical densities were recorded at 405 nm.

### 4.8. Superoxide Dismutase (SOD) Activity Assay

The Superoxide Dismutase Activity kit (Thermo Fisher), a colorimetric assay, was used for detection and quantification of the superoxide dismutase activity nanovesicle preparations. Samples were incubated for 20 min at room temperature after the addition of the sample and substrate and chromogenic detection reagent. The optical densities were recorded at 450 nm.

### 4.9. Nanovesicles Lysis

Nanovesicles were isolated from fruit through the abovementioned protocol. Then, they were counted by NTA and 10^11^ nanovesicles were resuspended in CHAPS buffer (Tris 10 mM pH 7.4, MgCl_2_ 1 mM, ethyleneglycoltetraacetic acid (EGTA) 1 mM, CHAPS 0.5%, glycerol 10%, phenylmethylsulfonyl fluoride (PMSF) 1 mM, leupeptin 1 µg/mL, pepstatin A 1 µg/mL, aprotinin 1 µg/mL and PMSF 1 mM).

For physical lysis, 10^11^ nanovesicles were sonicated in an ultrasonic bath sonicator at the maximum frequency (50 kHz) for two minutes.

Sample were analyzed through NTA (Malvern, Worcestershire, UK) to evaluate the number and size, and through a colorimetric ELISA kit to quantify the Total Antioxidant Capacity (JaICA, Japan).

### 4.10. Statistical Analysis

Results are reported as the means ± standard error (SE), and calculations were done using GraphPad Prism software (San Diego, CA, USA). An unpaired *t*-test (Student’s *t*-test) was applied to analyze the results. Statistical significance was set at *p* < 0.05.

## Figures and Tables

**Table 1:** Characterization of nanovesicles isolated from organic agriculture-derived and conventional agriculture-derived fruits.

Fruit	Type of Agriculture	Number of Nanovesicles	Mean (nm)
*A. chinensis*	Conventional Agriculture	9.0 × 10^11^ ± 3.9 × 10^10^	166.4 ± 3.9
	Organic Agriculture	1.1 × 10^12^ ± 2.7 × 10^10^	161.6 ± 0.6
*C. limon*	Conventional Agriculture	9.5 × 10^11^ ± 5.7 × 10^10^	210.0 ± 2.5
	Organic Agriculture	1.3 × 10^12^ ± 2.3 × 10^10^	193.5 ± 2.2
*C. paradisi*	Conventional Agriculture	3.6 × 10^12^ ± 4.8 × 10^10^	198.9 ± 3.7
	Organic Agriculture	5.0 × 10^12^ ± 7.8 × 10^10^	147.7 ± 2.4
*C. sinensis*	Conventional Agriculture	6.3 × 10^11^ ± 2.4 × 10^10^	201.7 ± 5.6
	Organic Agriculture	9.4 × 10^11^ ± 3.3 × 10^10^	178.8 ± 4.3

Data are expressed as the mean ± SE.

## Data Availability

The data presented in this study are available on request from the corresponding author.
